# Utility of the second-generation curcumin analogue RL71 in canine histiocytic sarcoma

**DOI:** 10.1007/s11259-023-10201-2

**Published:** 2023-08-19

**Authors:** Barnaby Kelly, Douglas Thamm, Rhonda J. Rosengren

**Affiliations:** 1 Department of Pharmacology and Toxicology, Unversity of Otago, 18 Frederick Street, Dunedin, 9085 New Zealand; 2 Flint Animal Cancer Center, Colorado State University, Fort Collins, CO USA

**Keywords:** Curcumin analogues, Canine histiocytic sarcoma, Cell cycle arrest and apoptosis

## Abstract

Canine histiocytic sarcoma is an aggressive cancer, with a high rate of metastasis. Thus, novel therapeutic approaches are needed. Synthetic analogues of curcumin have elicited potent anti-cancer activity in multiple in vitro and in vivo models of human cancer. Furthermore, the compound 3,5-bis(3,4,5-trimethoxybenzylidene)-1-methylpiperidine-4-one (RL71) has recently exhibited potent cell cycle arrest and apoptotic induction in a canine osteosarcoma cell line. To determine its potency in canine histiocytic sarcoma cells, cell viability of DH82 and Nike cells was measured using the sulforhodamine B assay. Flow cytometry was then used to analyse both cell cycle distribution and apoptosis. Of the five curcumin analogues examined, RL71, had the highest potency with EC_50_ values of 0.66 ± 0.057 µM and 0.79 ± 0.13 µM in the DH82 and Nike cell lines, respectively. Furthermore, RL71 at the 1x EC_50_ concentration increased G2/M cell cycle arrest 2-fold, and at the 2x EC_50_ concentration increased the number of apoptotic cells 4-fold. These findings are consistent with previous work using RL71 in both canine and human cancer cell lines. Future research should be directed on time-dependent changes, and mechanistic investigation in greater detail to elucidate RL71 mechanisms of action.

## Introduction

Histiocytic sarcoma (HS) is a condition that arises from malignant histiocytes infiltrating areas of the body, including the lungs, liver, spleen and lymph nodes. HS is separated into three major types: disseminated, localised and hemophagocytic HS (Fulmer and Mauldin 2007). HS occurs most frequently in the Bernese mountain dog (BMD), golden retriever, flat-coated retriever and Rottweiler breeds (Dobson 2013). It is a condition that is considered rare in dogs with the only clear hereditary link occurring in the BMD (Ruple and Morley 2016). HS is a very aggressive disease, with a median survival time as low as 49 days post-diagnosis (Abadie et al. 2009). This is due to a high likelihood of dissemination at the time of diagnosis (Abadie et al. 2009).

To date, no therapy has been specifically generated that targets HS in dogs. Current treatment practices commonly involve surgery where possible, followed with chemotherapy or stereotactic radiation therapy. Surgery and radiation therapy have been found to only effectively treat the condition for 398 and 240 days, respectively (Marconato et al. 2020). When chemotherapy treatment is used for HS common drugs include lomustine (CCNU), doxorubicin and vincristine (Moore et al. 2017). However, only CCNU has been reported to be a more effective treatment option increasing median survival times for localised HS to ~ 1 year post diagnosis (Rassnick et al. 2010). In the development of treatments for canine HS in the last 5 years there has been little advancements, with little long-term treatment options specifically aimed to target the more aggressive HS subtypes. Therefore, new treatment options are required.

One possible avenue of therapy is synthetic derivatives of curcumin, a polyphenolic flavonoid isolated from the *Curcumin longa* root, but limited research has been conducted in canine cancers (Anand et al. 2008). However, it was reported that curcumin elicited an EC_50_ value of 33 µM in DH82 canine histocytic carcinoma cells following 72 h of exposure. (Noronha et al., 2014). Additionally, in canine osteosarcoma cells the curcumin analogue 3,5-bis(3,4,5-trimethoxybenzylidene)-1-methylpiperidine-4-one (RL71) demonstrated potent anti-cancer activity through cell cycle arrest, regulation of cyclin proteins and induction of apoptosis through increases in cleave caspase − 3 (Kelly et al. 2023). Furthermore, a range of curcumin analogues have shown potency and efficacy in human breast and prostate cancer cells (Chen et al. 2018; Yadav et al. 2012a, b). However, to date no curcumin analogues have been tested in models of canine HS. Therefore, five curcumin analogues were screened for activity in HS cell lines. This was followed by cell cycle arrest and apoptosis analysis as proof of concept for their further use in canine HS research.

## Materials and methods

**Fig. 1:**
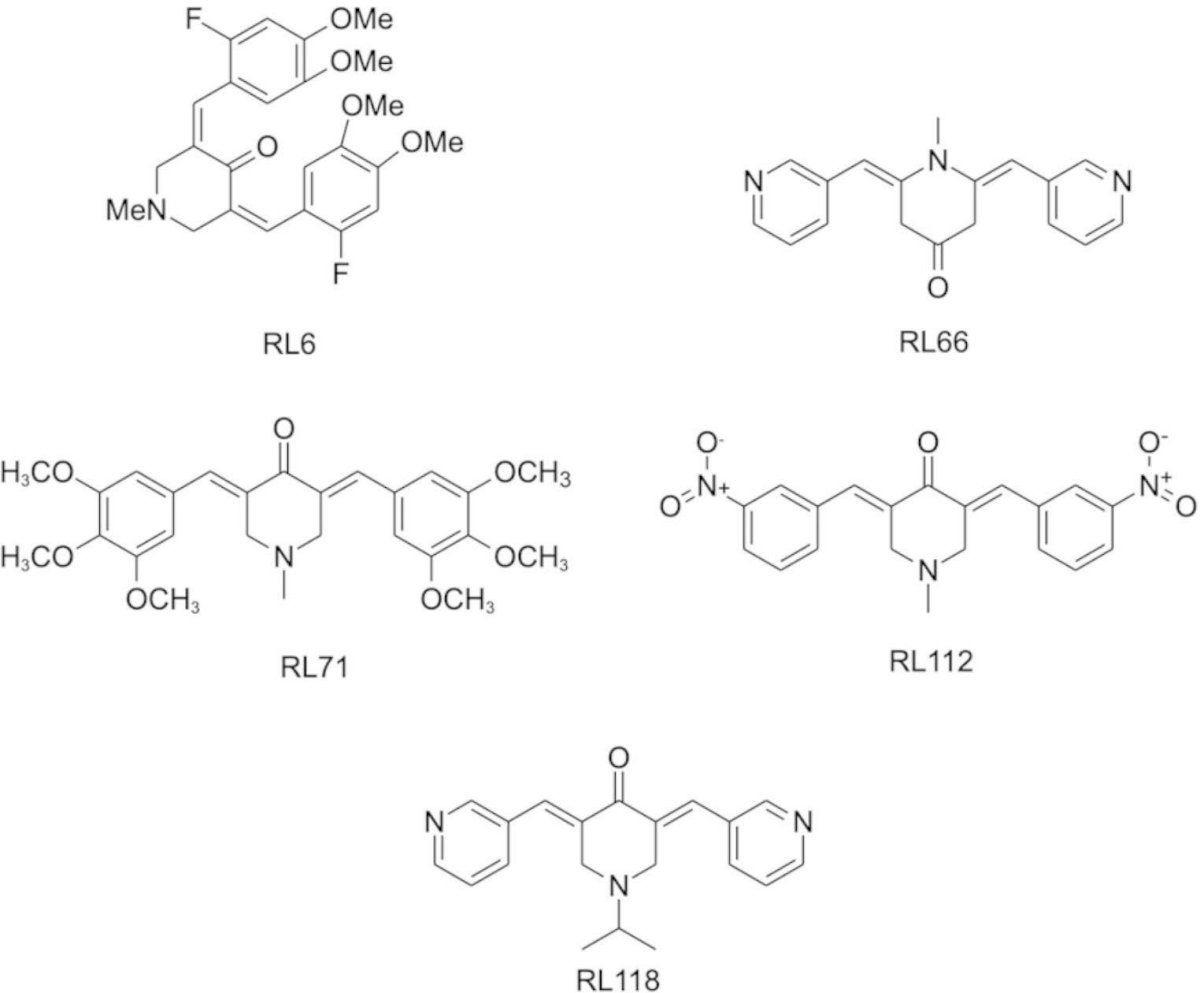
Chemical structure of curcumin analogues

*Materials.* DH82 HS cells were purchased from American Type Culture Collection (Manassas, VA, USA), and the donor dog Nike HS cell line was provided by Dr Douglas Thamm, Colorado State University. Propidium iodide (PI), dimethyl sulfoxide (DMSO), trichloroacetic acid (TCA), and sulforhodamine B salt (SRB) were purchased from Sigma Aldrich Ltd (Auckland, New Zealand). TRIS/HCL and acetic acid were purchased from Merck Life Sciences (Auckland, New Zealand). Annexin V APC was obtained from BD Pharmingen (San Jose, CA, USA). FxCycle PI RNase staining solutions were ordered from Life Technologies (Christchurch, New Zealand). Minimum essential medium Eagle (EMEM) with Earle’s balanced salts, 2 mM L-glutamine, penicillin (100 U/mL) and streptomycin (0.1 mg/mL) were purchased from Thermo Fisher Scientific (Auckland, New Zealand). The curcumin analogues (3E,5E)-3,5-bis(2-fluoro-4,5-dimethoxybenzylidene)-1-methylpiperidin-4-one (RL6), 1-methyl-3,5-bis[(E)-4-pyri-dyl)methylidene]-4-piperidone (RL66), RL71, 1-methyl-3,5-bis(3’-nitrobenzylidene)4-piperidone (RL112) and 1-isopropyl-3,5-bis[(pyridine-3-yl) methylene]piperidin-4-one (RL118) (Fig. 1) were gifted from Lesley Larsen (Department of Chemistry, University of Otago).

*Cell maintenance and validation*. DH82 and HS cells from the donor Nike were maintained in complete growth media composed of DMEM supplemented with 5% FBS, 1% penicillin/streptomycin and 1% glutamate at 37 °C, 5% CO_2_. Cell lines have been validated as of canine origin using multispecies multiplex PCR as described (O’Donoghue et al. 2011).

*Cytotoxicity*. DH82 cells (1 × 10^4^ cells/well) were seeded in 96 well plates and treated with a DMSO control (0.8%) or the curcumin analogues RL6, RL66, RL71, RL112 and Rl118 at concentrations ranging between 0.02 and 30 µM for 72 h. Nike cells (1 × 10^4^ cells/well) were treated with RL71 (0.02-30 µM) for 72 h. Cell number in each treatment well was determined using the SRB assay (Skehan et al. 1990). All results were obtained from three independent experiments conducted in triplicate.

*Cell cycle analysis*. Flow cytometry was used to analyse DNA content to determine cell cycle distribution. Nike HS cells were plated and treated with RL71 (0.5x EC_50_, 1x EC_50_ and 2x EC_50_) or a DMSO control (0.05%) for 24 h. Cells were harvested, washed with PBS, and then fixed in 70% ethanol. Following rehydration with PBS the cells were stained with propidium iodide (PI) and samples were analysed using a BD LSRFortessa flow cytometer and cell cycle distribution determined using Flow Jo software. Results are expressed as percent of cells in each phase of the cell cycle. To control for the events in the cell cycle analysis and apoptosis analysis using a flow cytometer, flow stability gating, pulse geometry gating and forward and side scatter gating was utilised. All results were obtained from three independent experiments conducted in triplicate.

*Induction of apoptosis.* Nike cells were seeded in 6-well culture plates at a density of 3 × 10^5^ and allowed to adhere for 24 h before treatment with RL71 (0.5x EC_50_, 1x EC_50_ or 2x EC_50_) or vehicle control (DMSO 0.05%) for 24 h. Apoptosis was assessed via co-staining cells with PI and Annexin V according to manufacturer instructions and imaged using a BD LSRFortessa flow cytometer. The number of apoptotic cells was determined via the use of Flow Jo software with results expressed as the percentage of apoptotic cells in each sample. All results were obtained from three independent experiments conducted in triplicate.

*Statistical analysis.* EC_50_ values were determined from a sigmoidal concentration-response (variable slope) equation via non-linear regression using Prism 9 software. Normality was examined using Shapiro-Wilk test (alpha = 0.05). Cell cycle distribution and apoptosis analysis was analysed using a two-way ANOVA coupled with a Tukey’s post-hoc test. For all statistical tests, p < 0.05 was the minimum requirement for a statistically significant difference.

## Results

**Fig. 2:**
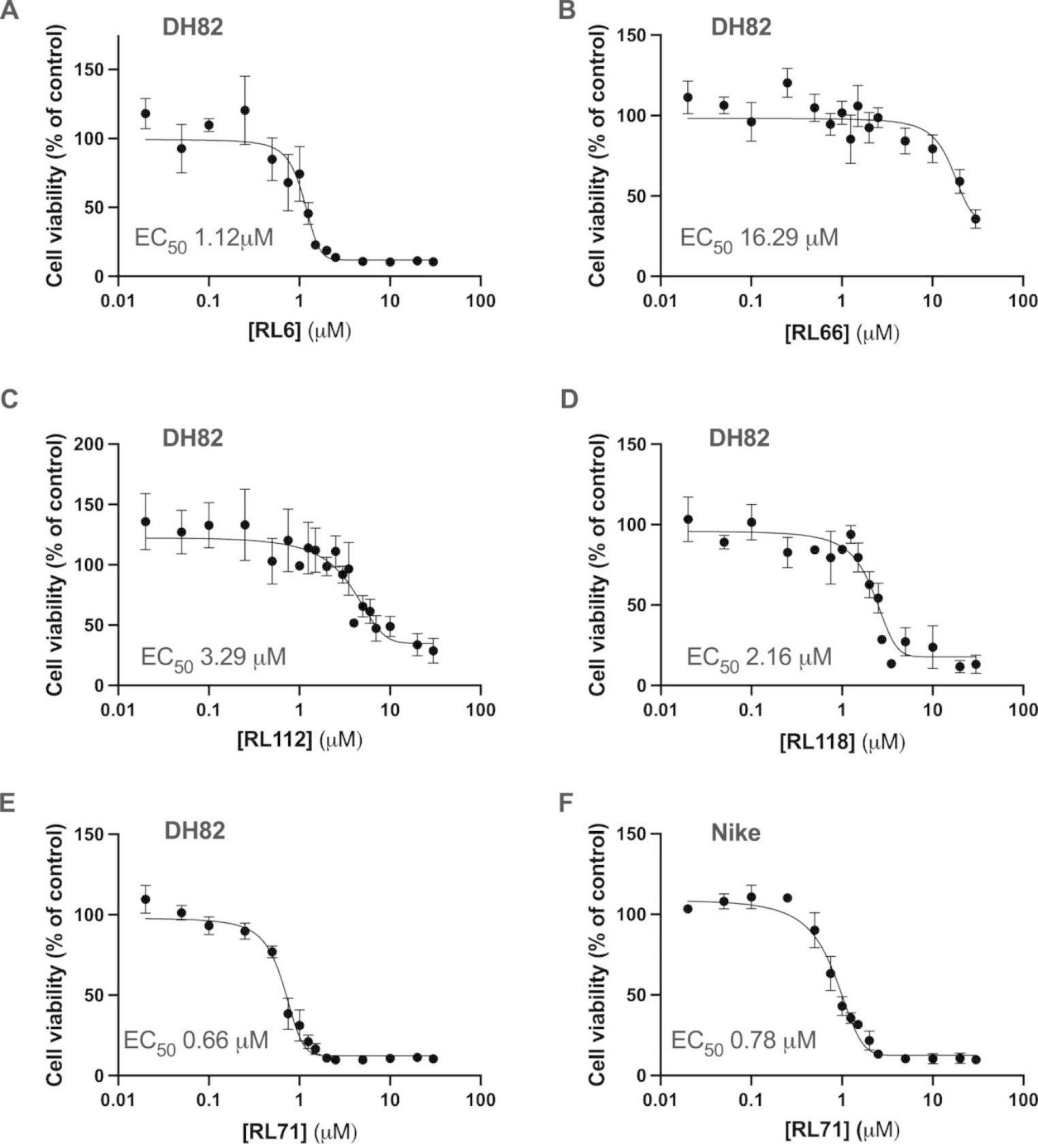
Effect of curcumin analogues on cell viability of canine DH82 and Nike histiocytic sarcoma cells. DH82 cells (**A**-**E**) and Nike (**F**) cells were seeded at a density of 1 × 10^4^ per well and then were treated with each curcumin analogue (0–30 µM) for 72 h (**A**) RL6, (**B**) RL66, (**C**) RL112, (**D**) RL118, (**E**) RL71 and Nike cells (1 × 10^4^ per well) were treated with (F) RL71. Cell viability was determined using the SRB assay with absorbance read at 510 nm and expressed as percentage of control. Results are expressed as mean ± SEM of three independent experiments performed in triplicate, EC_50_ values were determined using a sigmoidal concentration-response curve

Five curcumin analogues that elicited cytotoxicity in other human and canine cell lines were selected and screened for their potency in DH82 cells. Over 72 h of compound exposure at concentrations ranging from 0 to 30 µM, RL71 was the most potent curcumin analogue with an elicited EC_50_ value of 0.66 ± 0.057 µM (Fig. 2C). The rest of the analogues tested all elicited EC_50_ values ranging between 1.12 and 16.29 µM and were thus deemed to be less effective compared to RL71 (Fig. 2). RL71 was also examined in a second HS cell line (Nike). In these cells RL71 elicited an EC_50_ value of 0.79 ± 0.13 µM (Fig. 2F).

**Fig. 3:**
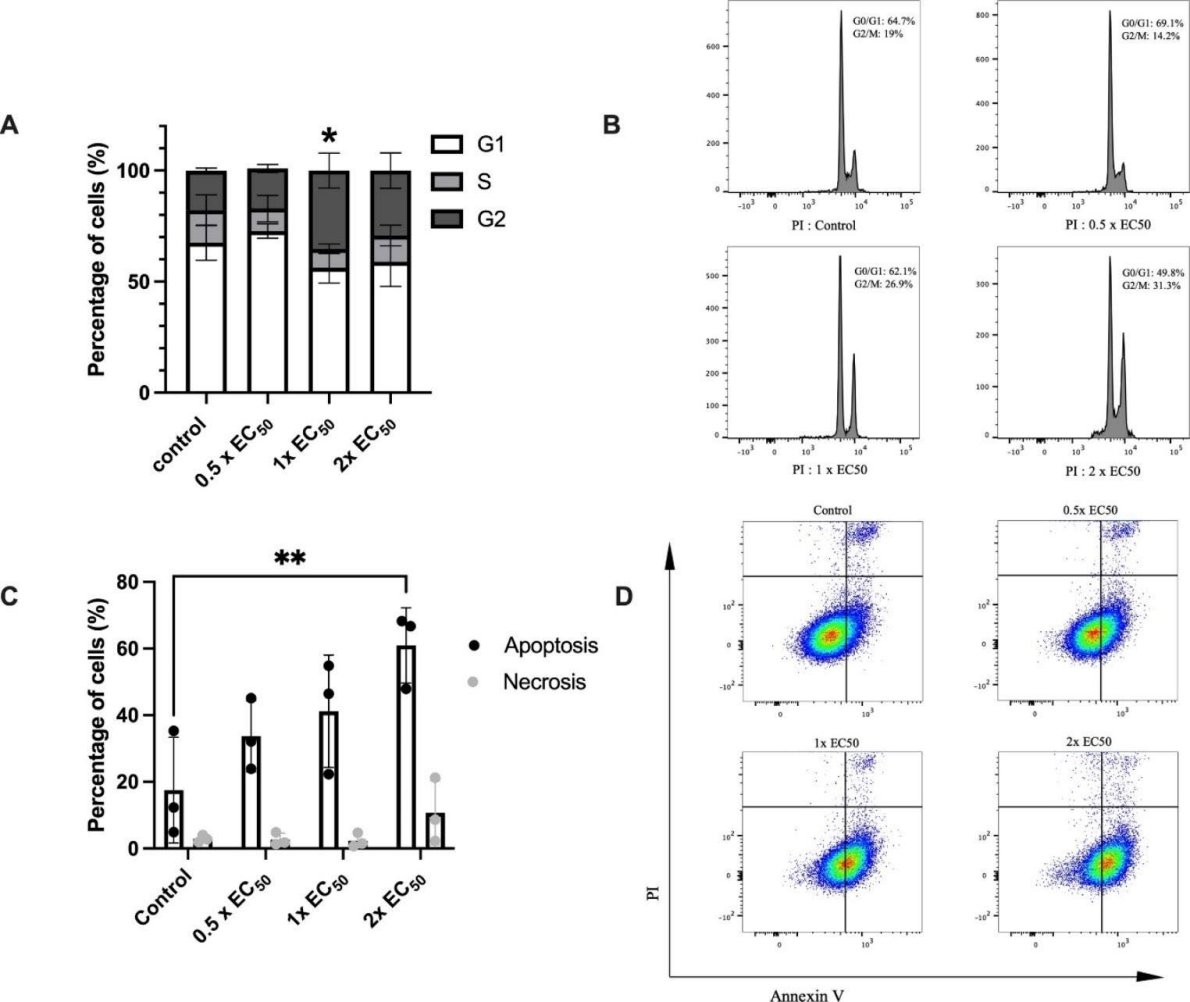
Effect of RL71 on cell cycle distribution and level of apoptosis in donor derived histiocytic sarcoma cells. Nike cells were seeded at a density of 3 × 10^5^ per plate and exposed to DMSO control (0.1%), 0.5x EC_50_, 1x EC_50_, 2x EC_50_ RL71 for 24 h. (**A**) Cell cycle distribution of Nike cells, (**B**) representative histogram of cell cycle distribution, (**C**) Percentage of apoptotic and necrotic cell distribution and (**D**) representative histogram of apoptotic distribution. Results are expressed as mean ± SEM of three independent experiments performed in triplicate. Statistical significance was determined via a two-way ANOVA coupled with a Tukey’s multiple comparison post-hoc test. *Statistically significant compared to control, p < 0.05 **Statistically significant compared to control, p < 0.005

These results confirmed RL71 as the lead compound and thus, it was examined to investigate its ability to modulate the cell cycle changes and modulate apoptosis. Compared to control, RL71 elicited the maximal increase of cells in the G2 phase by 97% above control at the 1x EC_50_ concentration (Fig. 3A). Comparatively, at the 2x EC_50_ concentration RL71 had the greatest effect on apoptosis and necrosis increasing both by 300% above control (Fig. 3C).

## Discussion

Canine HS is a rare, fatal condition, mainly effecting larger breeds such as the BMD (Dobson 2013; Ruple and Morley 2016). With limited therapeutic options available to dogs with this condition, the focus of this work was to identify new potential drug candidates in a pre-clinical model. Historically, curcumin and curcumin analogues have shown promising pre-clinical anticancer activity in various human cancer cell lines (Chen et al. 2018; Yadav et al. 2012b) as well as canine osteosarcoma cells (Kelly et al. 2023). In the present research, five curcumin analogues were examined for activity in canine HS cells.

The most potent compound was RL71, followed by RL6, which elicited an EC_50_ just above the desired sub micro-molar range. Comparatively, an analogue with a similar structure to RL71, RL66 produced a much higher EC_50_ of 16.29 µM. The main structural differences between RL71 and RL66 is the methoxy groups on carbons 2, 3 and 4 of its benzene rings. It appears that the presence of these groups enhanced the cytotoxicity of the compound against DH82 cells. Recently, a similar effect has been observed with pyrazole curcumin derivatives, where the most potent derivative tested had much greater stability compared to less potent compounds (Feriotto et al. 2023). Structurally, both the potent pyrazole derivative and RL71 are similar. The main characteristic difference is the lack of methoxy groups and addition of a nitrogen group added to a larger carbon backbone. When compared to RL6 and RL66, the inclusion of the methoxy groups in RL71’s structure increased bioavailability of a single oral dose of 8.5 mg/kg. Specifically, the peak plasma concentration in mice was 6-fold higher for RL71 compared to RL66, and G2/M phase cell cycle arrest was elicited at a 2-fold lower concentration in triple negative breast cancer cells (Yadav et al. 2012a, b).

Cell cycle progression and apoptosis was investigated to elucidate how RL71 mediated the reduction in cell viability. Exposure to RL71 significantly increased both G2/M arrest and apoptosis however, this effect occurred at different concentrations. Cell cycle arrest was elicited at a lower concentration of 0.79 µM, whereas 1.58 µM was required to increase apoptosis. It was observed that G2/M cell populations also increased at the higher concentration, however this effect was not significant. A possible reason for this could be that the action of RL71 at higher concentrations led to increased necrosis and less viable cells even following 24-hour exposure due to quicker onset. These findings are consistent with previous uses of RL71 that demonstrated both an increase in G2/M arrest and apoptosis (Yadav et al. 2012b). There is presently no published work investigating the mechanism of curcumin/curcumin analogues in canine HS, but comparisons can be made to other canine sarcomas. Firstly, in human head and neck squamous cell carcinomas, curcumin (15 µM) and the curcumin analogue WZ37 (25 µM) increased G2/M phase arrest following 48 h of exposure (Zhang et al. 2020). Furthermore, a curcumin pyrazole derivative has also triggered significant G2/M accumulation and apoptosis in MG63 human osteosarcoma cells at concentrations of 2.7 µM (Feriotto et al. 2023). In a recent paper, RL71 increased both G2/M cell cycle arrest and apoptosis at concentrations greater than 0.76 µM in canine osteosarcoma cells, and elicited sub-micromolar EC_50_ values (Kelly et al. 2023). Thus, further research with RL71 is warranted in canine cancers and should include an analysis of both concentration and tine-dependent effects. Since RL71 is more bioavailable than curcumin, non-toxic (Yadav et al. 2012b), and more effective at regulating the cell cycle compared to other curcumin analogues, it is a promising compound for future investigations in dogs with cancer. In a xenograft model of triple negative breast cancer, bioavailability, tumor suppression and tumor targeting were improved through delivery of RL71 in the form of styrene maleic acid (SMA) micelles (Martey et al. 2017). Thus, any future of RL71 is likely to be as a nanomedicine.

In summary, RL71 appears to be an interesting drug candidate for canine HS. Future studies involving RL71 in canine HS should focus on time-dependent effects as well as investigation into the p53 pathway, given its mutation profile in canine HS (Hedan et al. 2011). Further research with RL71 is warranted as it has already been proven safe in mice following 10 weeks of daily oral dosing (8.5 mg/kg) (Yadav et al. 2012b) as well as weekly IV dosing (10 mg/kg) for 90 days as a nanoformulation (SMA-RL71) (Martey et al. 2017) Furthermore, RL71 is more bioavailable compared to curcumin (Anand et al. 2007). Overall, RL71 is a potent cytotoxic compound that is both safe and bioavailable in vivo. These factors warrant further investigation in canine models especially given the need for drug treatments for canine HS. Once mechanisms of action are determined in cell models of canine cancer, there is the potential for RL71 to be developed as a nanomedicine for a range of canine cancers that currently need novel treatments.

## Data Availability

All datasets used and analysed during the current study protocol are available from the corresponding author on reasonable request.
